# A Massive Chondroblastoma in the Proximal Humerus Simulating Malignant Bone Tumors

**DOI:** 10.1155/2013/673576

**Published:** 2013-03-25

**Authors:** Ichiro Tonogai, Mitsuhiko Takahashi, Hiroaki Manabe, Toshihiko Nishisho, Seiji Iwamoto, Shoichiro Takao, Seiko Kagawa, Eiji Kudo, Natsuo Yasui

**Affiliations:** ^1^Department of Orthopedics, Institute of Health Biosciences, The University of Tokushima, Japan; ^2^Department of Radiological Science, Institute of Health Biosciences, The University of Tokushima, Japan; ^3^Department of Human Pathology, Institute of Health Biosciences, The University of Tokushima, Japan

## Abstract

Chondroblastoma is a mostly benign bone neoplasm that typically affects the second decade of life and exhibits a lytic lesion in the epiphysis of long bones. We report an extreme case of massive, destructive chondroblastoma of the proximal humerus in a 9-year-old girl. It was difficult to differentiate using imaging information the lesion from malignant bone tumors such as osteosarcoma. Histopathological examination from biopsy proved chondroblastoma. The tumor was resected after preoperative transcatheter embolization. Reconstructive procedure for the proximal humerus was not performed due to the local destruction. The present case demonstrates clinical and radiological differentiations of the massive chondroblastoma from the other lesions and histopathological understandings for this lesion.

## 1. Introduction

Chondroblastoma is a mostly benign bone neoplasm that typically affects the second decade of life and exhibits a lytic lesion in the epiphysis of long bones [1, 2]. Pain localized to the lesion is a common clinical symptom of chondroblastoma, where patients present with local tenderness and swelling [1–3]. However, a large chondroblastoma that compromises neighboring joint is occasionally encountered, where clinical and radiological features are not well known. We report here a case of a massive, destructive lesion of chondroblastoma in the proximal humerus, which provides clinical, radiological, and histopathological significances of this lesion. 

## 2. Case Report

The patient and her family were informed that data concerning her case would be submitted for publication, and they provided written consent.

**Figure 1:**
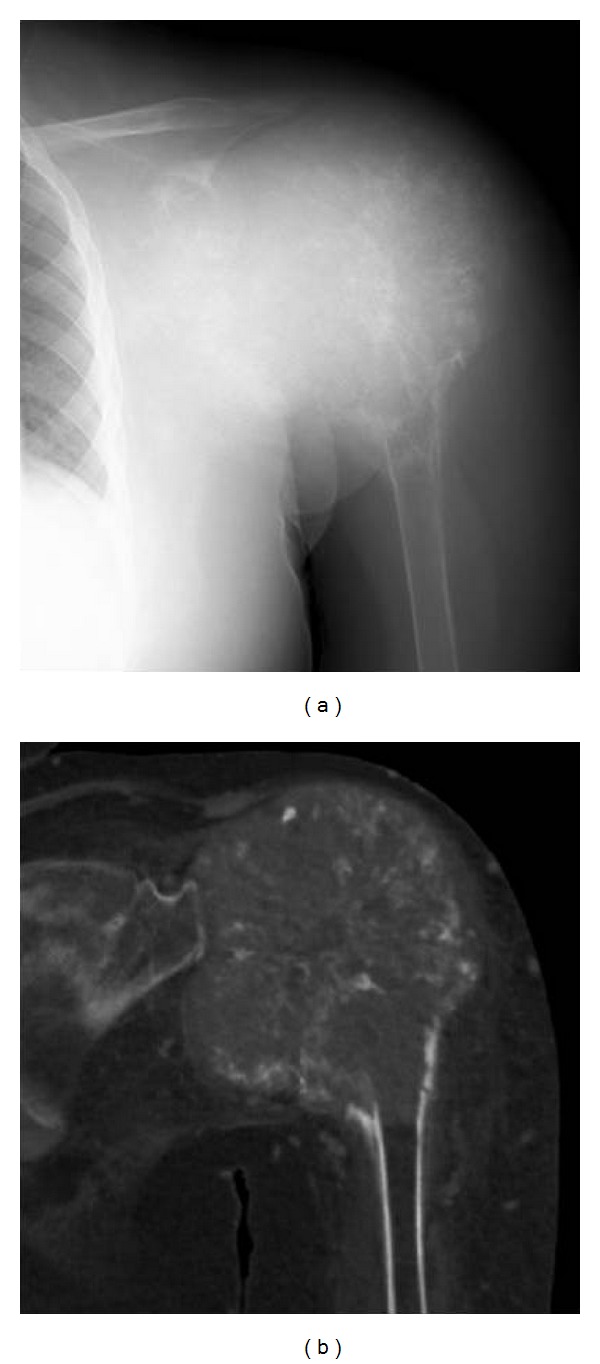
Radiograph (a) and CT reconstruction (b) showed an expanded, destructive lesion of the proximal humerus containing irregular calcifications. There was almost no cortical margin around the lesion.

**Figure 2:**
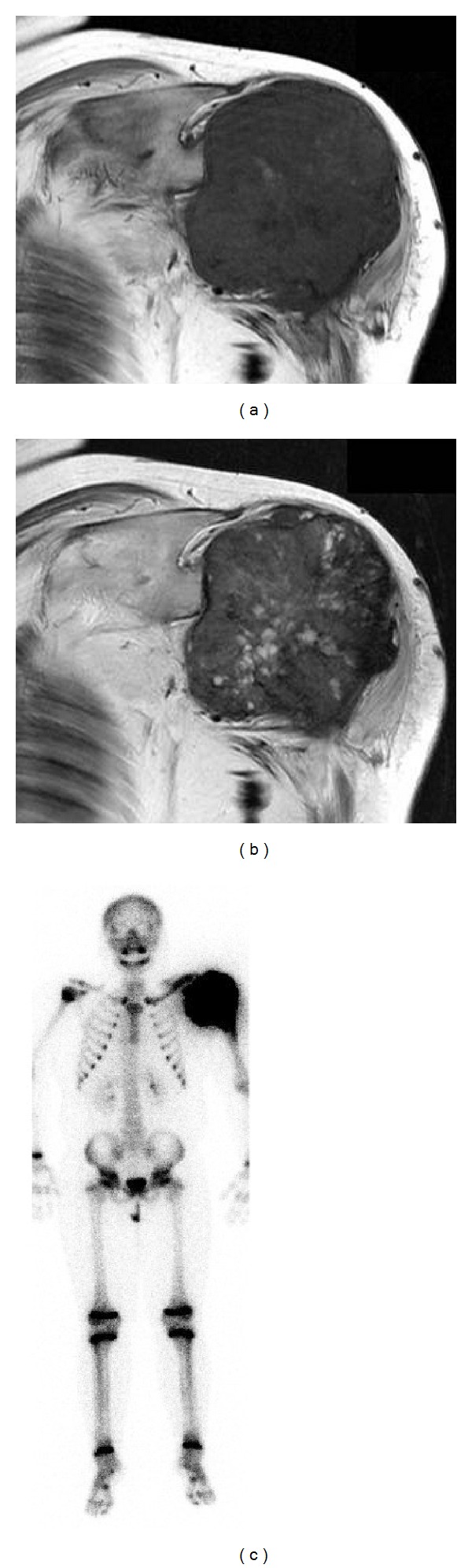
Coronal T1-weighted (a) and T2-weighted (b) MR images demonstrated a huge tumor in the proximal humerus. Whole body technetium-99m scan showed abnormal uptake in the left proximal humerus and surrounding area (c).

A 9-year-old girl who had been treated for adjustment disorder was referred to our hospital for a massive lesion of the left shoulder with severe limitation of joint mobility over approximately one year. The left shoulder exhibited local warmth and varicoses. Neither motor, sensory, nor circulatory disorder was seen distal to the shoulder joint. Laboratory tests showed moderate increases in C-reactive protein (CRP, 2.04 mg/dL) and alkaline phosphatase (817 U/L). Plain radiographs showed a 14 × 15 cm poorly-demarcated mass with irregular calcification (Figure 1(a)). Computed tomography (CT) scans demonstrated a 13 × 15 × 15 cm hypodense mass containing segmented calcifications (Figure 1(b)). On T1-weighted magnetic resonance imaging (MRI), the mass showed mostly a low-intensity signal under the acromion and the thinned deltoid muscle (Figure 2(a)). On T2-weighted imaging, the lesion had multiple high-intensity nodules within the low-intensity mass (Figure 2(b)). A bone scan revealed extremely high uptake by the lesion and the surrounding clavicle and glenoid (Figure 2(c)). No mass lesion was detected in the lungs on either CT or bone scan. 

**Figure 3:**
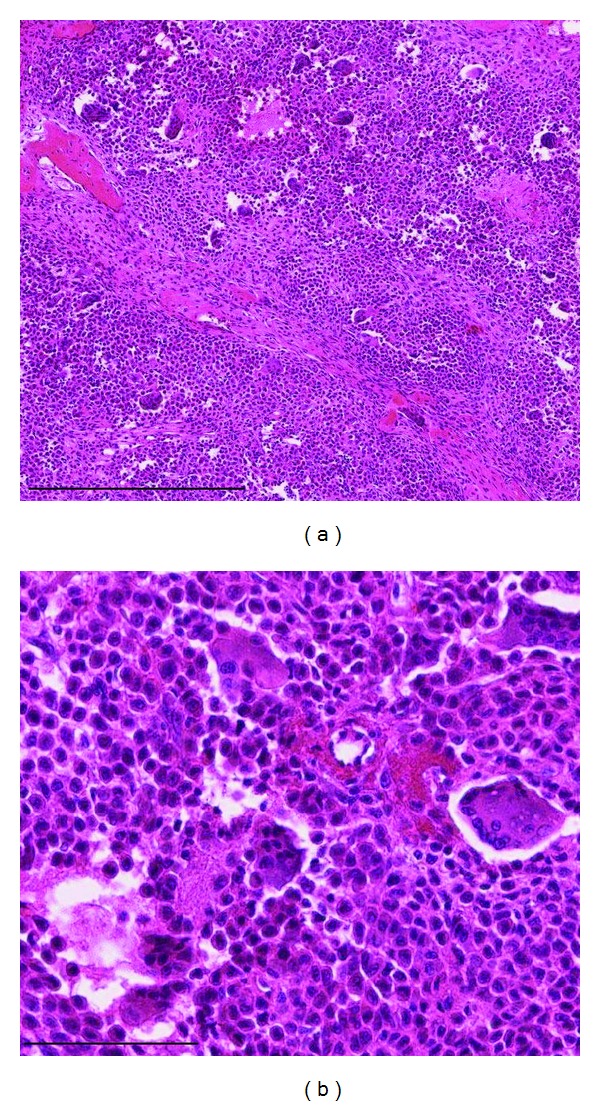
Low (a) and high (b) magnifications of hematoxylin and eosin staining. Scale bars indicate 500 *μ*m (a) and 100 *μ*m (b), respectively.

**Figure 4:**
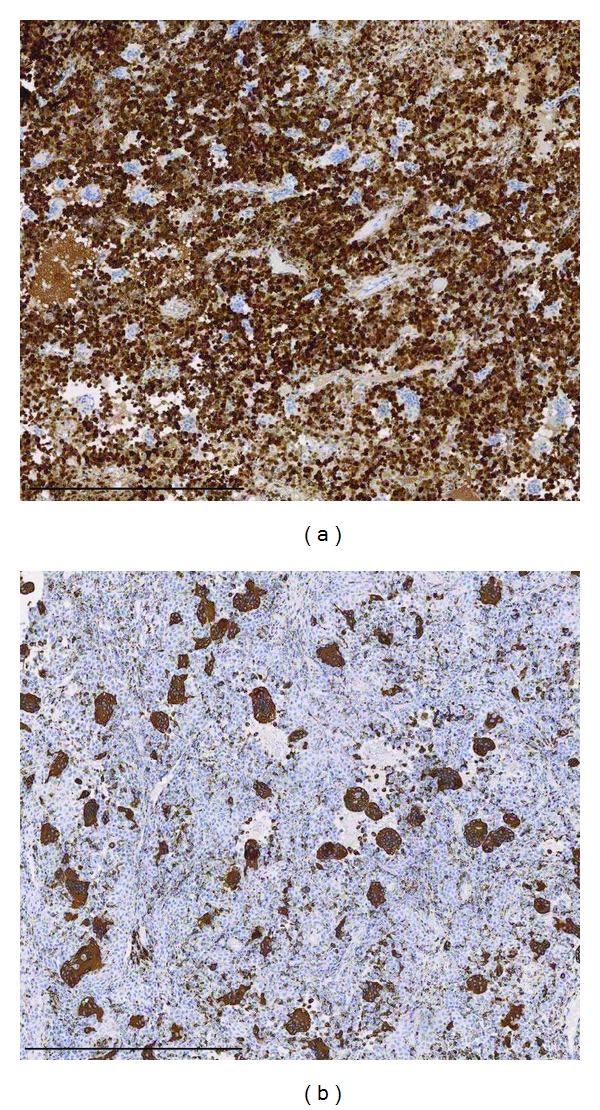
Immunohistochemical staining for S-100 protein (a) and for CD68 (b). Both scale bars indicate 500 *μ*m.

As malignant bone tumor such as osteosarcoma was a plausible diagnosis, incisional biopsy was performed. The lesion had a thin membrane but not a firm cortical bone structure. The specimen grossly consisted of a white, solid component and a grey, soft fibrocartilaginous element. Histological examination revealed homogeneous spread of round- to polygonal-shaped mononuclear cells having an eosinophilic cytoplasm and a round- to ovoid-shaped nucleus (Figure 3). These hypercellular elements were separated by fragmented bony trabeculae and interstitial fibrocartilaginous matrices. Multinucleated giant cells were disseminated, and vascular cavities were also abundant. Immunohistochemical examination showed strong positive staining for S-100 protein in mononuclear cells, and for CD68 (PGM1), which is a marker of histiocytes, in multinuclear giant cells and some mononuclear cells (Figure 4). The Ki-67 labeling index was approximately 10% of the mononuclear cells. The histopathological and immunohistochemical findings were typical for a chondroblastoma, and no evidence of malignancy such as a chondroblastic osteosarcoma was noted.

**Figure 5:**
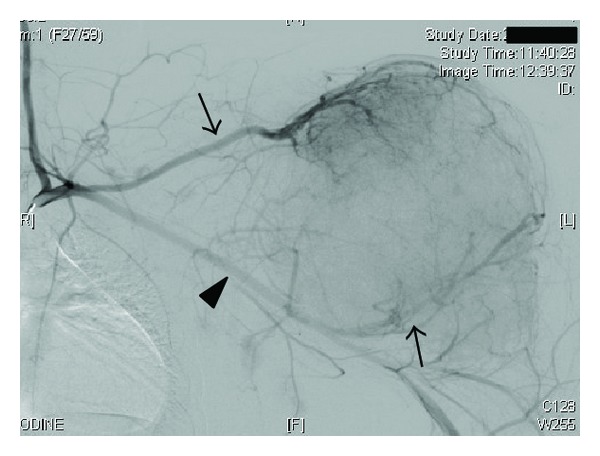
Preoperative angiography at the level of the left subclavicular artery showing two enlarged arteries (arrows) reached upper and lower poles of the lesion, respectively. Note diameters of these feeding arteries compared with the axillar artery (arrowhead).

Follow-up CT revealed multiple nodular lesions over the bilateral lungs, which could indicate spontaneous transformation to aggressive or malignant chondroblastoma with the potential to metastasize [4, 5]. These were later concluded to be nodular septic embolisms from inflammatory signs and were successfully managed with antibiotics. Considering the local varicoses and abundant vessels on the histological section, preoperative transcatheter arterial embolization was performed to minimize intraoperative hemorrhage. Angiography revealed the hypervascular lesion fed by the enlarged suprascapular and humeral circumflex arteries (Figure 5). Afterwards, *en bloc *tumor resection via an extended deltopectoral incision was completed. The resected specimen measured 18 × 15 × 12 cm and weighed 2.4 kg, containing fibrocartilaginous, calcified, and hemorrhagic components. Total amount of bleeding during operation was approximately 2,300 mL. Since all muscles inserting the proximal humerus and the articular surface of the glenoid were completely compromised, shoulder joint reconstruction was not performed. The axillary and radial nerves were released from the tumor, although both had been stretched by expansion of the tumor. The humeral insertion of the thinned deltoid and the long head of triceps were preserved, and the short head of biceps, which was detached at the beginning, was repositioned. The entire specimen was sliced parallel in the coronal plane, and the whole of the largest slice was examined thoroughly. Histopathological findings of the resected specimen were consistent with those of the previous biopsy findings. No atypical cells, which indicate malignant transformation of the tumor, were found anywhere. The specimens were further examined by a consultant specialist of sarcoma pathology, and the definitive diagnosis of chondroblastoma was confirmed. 

**Figure 6:**
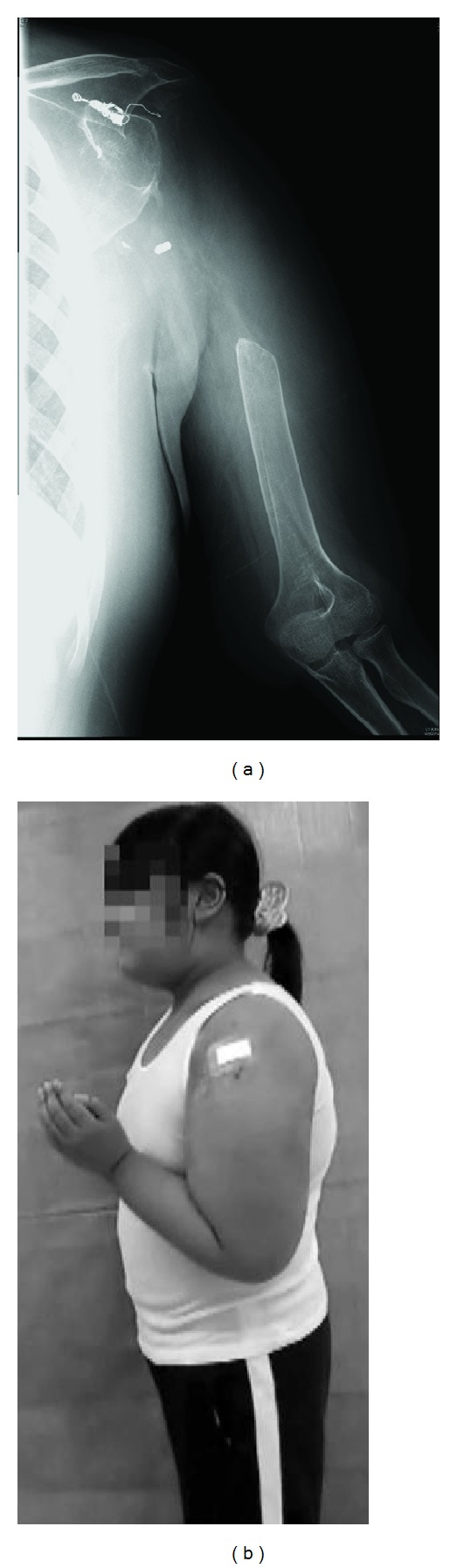
One-year postoperative radiograph showing the proximal humerus was resected and coils for preoperative embolization are left (a). Postoperative appearance of the left arm showing active elbow flexion (b).

Two years after surgery, the patient had readjusted to school life, despite complete loss of shoulder mobility. No recurrence or metastasis has been detected (Figure 6(a)). The patient uses her left arm as a supportive limb, with up to chest level of hand positioning, no limitation of manual dexterity, and minor load of lifting ability (Figure 6(b)). The Musculoskeletal Tumor Society (MSTS) score [6] is 21/30. 

## 3. Discussion

Chondroblastoma is recognized as a benign, cartilaginous lesion that characteristically arises in the epiphysis of long bones, particularly the humerus, tibia, and femur [1] and that represents less than 1% of all primary bone tumors [2]. It was first described as a distinct entry in 1942 by Jaffe and Lichtenstein [7]. Since chondroblastoma usually causes pain localized to the lesion, where patients present with local tenderness, swelling, and limitations of neighboring joint movement [3], it is possible to be identified before it grows beyond the cortex. Thus, the primary procedure for chondroblastoma is curettage followed by bone grafting. To the best of our knowledge, only two cases of huge chondroblastoma have been reported in the English literature. Nakatani and Beppu [8] conducted curettage followed by autologous and artificial bone graft for an expansive chondroblastoma of the proximal humerus. Ozkoc et al. [9] performed* en bloc* resection of an intra-articular giant tumor arising from the scapula, which measured 39 × 30 × 18 cm  and weighed 14.44 kg. 

In the present case, the rotator cuff and the articular surface of the glenoid were completely destroyed, which made functional reconstruction of the shoulder joint difficult. The distal half of the humerus is, then, floating with intact wrist and hand functions. Suspension arthroplasty of the shoulder using a vascularized fibula graft should be one of the reconstructions following a massive defect of the proximal humerus. To date, the patient has not received reconstructive procedures, considering her psychiatric background, and neither the patient nor her family is open to discussing reconstructive surgery. 

The radiological feature of chondroblastoma is typically a lytic lesion arising in the epiphysis with an eccentric location, mostly without periosteal reaction [1–3]. The lesion usually has a thin sclerotic border and sometimes crosses the physis. However, accurate diagnosis of overgrown chondroblastoma is difficult using only imaging information [8, 9], because overgrown chondroblastoma has a profile that resembles the chondroblastic osteosarcoma, which constitutes approximately 25% of the osteosarcoma category [10]. Metaphysis origin, bone-forming tumor matrix, aggressive periosteal reaction, and young patient age should be considered as signs of latent chondroblastic osteosarcoma [11]. In the present case, MRI revealed the presence of a lobular structure of high signal intensity on T2-weighted images. Such images reflected histologically cartilage components including the coexistence of a bone- and cartilage-forming tumor matrix [12]. Differentiation of chondroblastic osteosarcoma from chondroblastoma was significant in terms of treatment and prognosis. It should be noted that accurate diagnosis could be made by appropriate biopsy. 

In the present case, the pulmonary lesions were concluded to be nodular septic embolisms but not metastatic lesions. Pulmonary metastases are common in osteosarcoma. It would be rare for such a huge primary lesion not to show any pulmonary metastases if osteosarcoma had been present over approximately one year. Pulmonary metastases have been reported even in the cases of chondroblastoma, and these metastases were generally observed after operative managements of the primary lesion [3–5, 9]. An unusual case of chondroblastoma metastasizing in the lung before surgical intervention was previously reported [13]. Although preoperative arterial embolization could be beneficial to prevent further hemorrhage considering the enlarged vessels and hypervascularity of the lesion, tumor embolization was reported to increase the possibility of chondroblastoma metastasis [14]. The period from the diagnosis of primary tumor to detection of lung metastasis was on average 8.4 years. Thus, long-term observation for metastasis should be required for the present case.
